# Effects on quality of life, anti-cancer responses, breast conserving surgery and survival with neoadjuvant docetaxel: a randomised study of sequential weekly versus three-weekly docetaxel following neoadjuvant doxorubicin and cyclophosphamide in women with primary breast cancer

**DOI:** 10.1186/1471-2407-11-179

**Published:** 2011-05-18

**Authors:** Leslie G Walker, Jennifer M Eremin, Mark M Aloysius, Wichai Vassanasiri, Mary B Walker, Mohamed El-Sheemy, Ged Cowley, Jeanette Beer, Srila Samphao, Janice Wiseman, Jibril A Jibril, David Valerio, David J Clarke, Mujahid Kamal, Gerald W Thorpe, Karin Baria, Oleg Eremin

**Affiliations:** 1 Oncology Health Centres and the Institute of Rehabilitation, University of Hull, Kingston upon Hull, East Riding of Yorkshire HU3 2PG, UK; 2 Department of Clinical Oncology, Lincoln County Hospital, Lincoln LN2 5QY, UK; 3 Research & Development Department, Lincoln County Hospital, Lincoln, UK; 4 Lincoln Breast Unit, Lincoln County Hospital, Lincoln, UK; 5 Department of Health, Life and Social Sciences, University of Lincoln, Lincoln, LN6 7TS, UK; 6 Department of Pathology, Lincoln County Hospital, Lincoln, UK; 7 Department of Radiology, Lincoln County Hospital, Lincoln, UK; 8 Department of Surgery, Nottingham University Hospitals, Nottingham NG7 2UH, UK

**Keywords:** Breast cancer, Docetaxel, Neoadjuvant therapy, Quality of life

## Abstract

**Background:**

Weekly docetaxel has occasionally been used in the neoadjuvant to downstage breast cancer to reduce toxicity and possibly enhance quality of life. However, no studies have compared the standard three weekly regimen to the weekly regimen in terms of quality of life. The primary aim of our study was to compare the effects on QoL of weekly versus 3-weekly sequential neoadjuvant docetaxel. Secondary aims were to determine the clinical and pathological responses, incidence of Breast Conserving Surgery (BCS), Disease Free Survival (DFS) and Overall Survival (OS).

**Methods:**

Eighty-nine patients receiving four cycles of doxorubicin and cyclophosphamide were randomised to receive twelve cycles of weekly docetaxel (33 mg/m^2^) or four cycles of 3-weekly docetaxel (100 mg/m^2^). The Functional Assessment of Cancer Therapy-Breast and psychosocial questionnaires were completed.

**Results:**

At a median follow-up of 71.5 months, there was no difference in the Trial Outcome Index scores between treatment groups. During weekly docetaxel, patients experienced less constipation, nail problems, neuropathy, tiredness, distress, depressed mood, and unhappiness. There were no differences in overall clinical response (93% vs. 90%), pathological complete response (20% vs. 27%), and breast-conserving surgery (BCS) rates (49% vs. 42%). Disease-free survival and overall survival were similar between treatment groups.

**Conclusions:**

Weekly docetaxel is well-tolerated and has less distressing side-effects, without compromising therapeutic responses, Breast Conserving Surgery (BCS) or survival outcomes in the neoadjuvant setting.

**Trial registration:**

ISRCTN: ISRCTN09184069

## Background

Neoadjuvant chemotherapy (NAC) is being used with increasing frequency in the treatment of patients with locally advanced breast cancers (LABCs) [1]. It has been considered for operable breast cancer in order to downstage the disease and enable breast-conserving surgery (BCS) to be carried out [2,3]. NAC may deal with occult micrometastases, thereby, improving survival [4].

The National Surgical Adjuvant Breast and Bowel Project (NSABP) B-18 study comparing anthracycline-based chemotherapy preoperatively with the same regimen postoperatively has shown an enhanced rate of BCS with NAC [5]. No survival difference was seen between both groups. Other studies, including a recent meta-analysis, have also demonstrated comparable results [6-8]. Therefore, NAC can increase BCS rate, but the effect on long-term survival remains unproven.

The optimal NAC schedule is unknown. Several studies have shown promising results of using taxanes following anthracyclines, particularly in terms of enhancing a pathological complete response (pCR) rate, a surrogate marker of long-term survival [6,9-11]. Nevertheless, NAC is associated with significant morbidity and reduced quality of life (QoL) [12,13].

Studies of weekly docetaxel in metastatic breast cancer have demonstrated significantly reduced toxicity profiles, while maintaining a level of efficacy comparable with the 3-weekly regimen [14-16]. A phase II study of weekly docetaxel alone as NAC has shown a high pCR rate with less haematological toxicity [17]. A randomised NAC study comparing weekly versus 3-weekly paclitaxel followed by 4 cycles of 5-fluorouracil, doxorubicin, and cyclophosphamide has confirmed a superiority of the weekly schedule in enhancing a pCR rate [18]. Recently, the results from the Intergroup Trial E1199 comparing paclitaxel or docetaxel given preoperatively every 3 weeks or weekly following doxorubicin and cyclophosphamide in operable breast cancer have demonstrated no differences in disease-free survival (DFS) between taxanes and schedules. However, DFS was significantly improved with weekly paclitaxel and 3-weekly docetaxel, compared with 3-weekly paclitaxel [19].

The primary aim of our study was to compare the effects on QoL of weekly versus 3-weekly sequential neoadjuvant docetaxel. Secondary aims were to determine the clinical and pathological responses, incidence of Breast Conserving Surgery (BCS), Disease Free Survival (DFS) and Overall Survival (OS).

## Methods

### Patient eligibility

Women (ages 18-70 years) presenting to the Lincoln Breast Unit were invited to participate if they had unilateral/bilateral large (≥3 cm) or LABCs (T3, T4, TxN2), no distant metastases; WHO performance status of <2; no history or evidence of abnormal cardiac function; adequate haematological, renal, and hepatic function; and were not pregnant.

Exclusion criteria were a previous malignancy (except curatively treated carcinoma in situ of the cervix or basal cell carcinoma of skin); previous cytotoxic, endocrine, or radiotherapy; active infection; contraindications to corticosteroid administration; pre-existing neurotoxicity (> grade 2) (NCI-CTC); significant cognitive impairment or dementia, and inability to complete QoL questionnaires or provide informed consent.

The study protocol was approved by the Research Ethical Committee. Patients provided signed informed consent.

### Study design

Diagnosis was established by examination (calliper measurements), mammography, ultrasonography, and core needle biopsy. All women underwent a chest radiograph, bone scintigraphy and liver ultrasonography to exclude metastases before entry.

**Figure 1:**
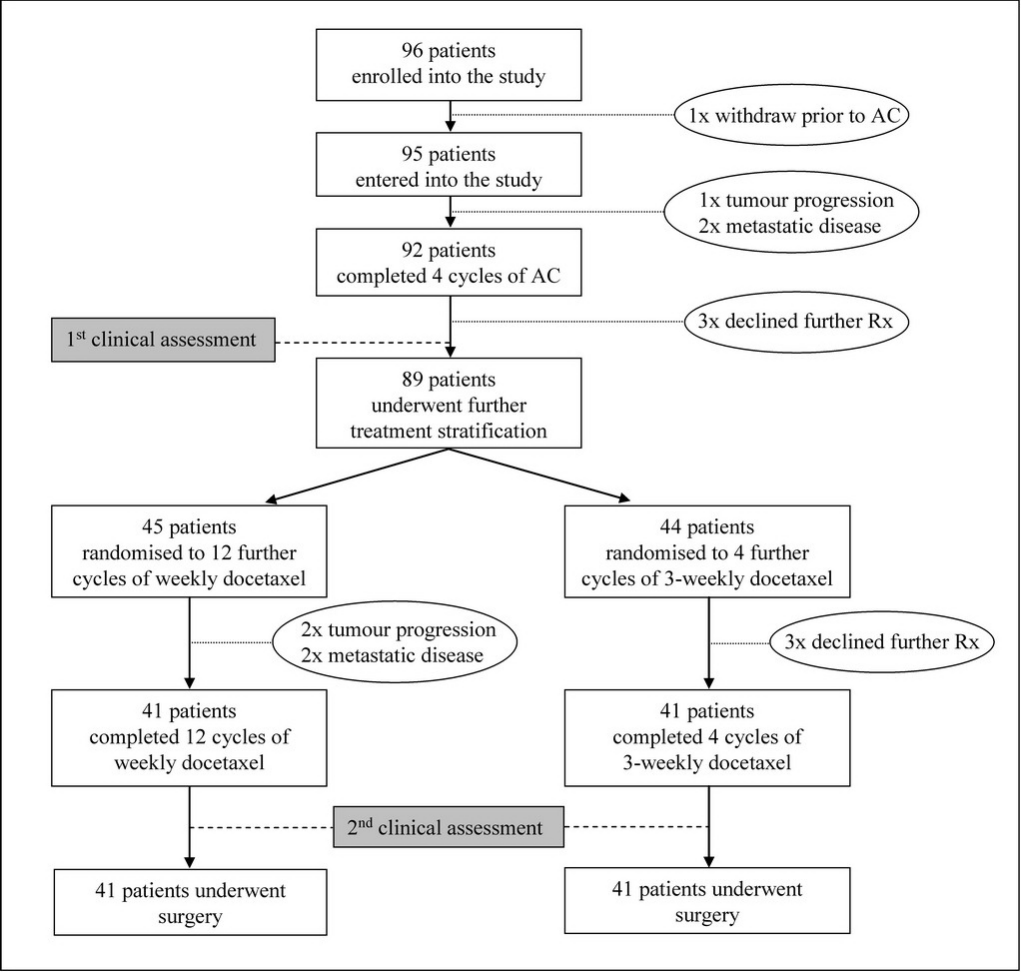
**Schematic diagram of patient entry, subsequent randomisation and treatment regimens**. Rx, treatment

Patient entry, participation, randomisation and treatment are shown schematically in Figure 1. Randomisation was carried out using permuted blocks (Instat 2). Treatment allocation was performed independently by a third party, by opening sealed sequenced envelopes containing treatment allocation. Tumour progression (local or distant) on treatment resulted in withdrawal.

QoL was assessed before randomisation, every 3 weeks during docetaxel, and 3 weeks after completion of chemotherapy.

Tumour responses were assessed using calliper and ultrasound measurements. Wide local excision or mastectomy was performed with either sample or clearance of the axillary lymph nodes. Pathological responses of the tumour in the breast and metastatic lymph nodes were assessed and graded. Patients having BCS received radiotherapy to the breast. If sampling established metastases, axillry and supraclavicular nodes were treated by radiotherapy. Patients who underwent mastectomy received radiotherapy to the chest wall if deemed at risk of local recurrence. Tamoxifen was given to patients whose tumours had receptors for oestrogen (ER) and/or progesterone (PR).

### Treatment regimen

Patients received intravenous injections of doxorubicin (A) 60 mg/m^2 ^and cyclophosphamide (C) 600 mg/m^2 ^every 3 weeks for four cycles. The weekly group were given twelve further cycles of docetaxel, 33 mg/m^2 ^as one-hour intravenous infusions at weekly intervals with a two-week break between cycle 6 and 7. Patients in the 3-weekly group received four further cycles of docetaxel at a dose of 100 mg/m^2^, as one-hour intravenous infusions every 3 weeks. Patients received dexamethasone and ondansetron, before and after therapy. Both groups received identical total doses of docetaxel and steroids.

If the nadir neutrophil count on day 21 was <1.5 × 10^9^/L, with or without fever, or the platelet count <100 × 10^9^/L, the subsequent doses (A, C, or docetaxel) were reduced by 25%; dose reductions were maintained during subsequent cycles. For a second episode of grade IV neutropenia, the subsequent doses of chemotherapy were reduced by 50%. Granulocyte colony-stimulating factor (G-CSF) was given to patients with neutropenia-associated sepsis. A delay of more than two weeks for haematological recovery necessitated taking the patient off study.

### Surgery

Patients underwent surgery 4 weeks after the last cycle of chemotherapy; surgery was performed earlier if there was tumour progression or prolonged toxicity. The type of surgery carried out depended on the response to chemotherapy, the amount of residual tumour, and patient preference. Axillary surgery was performed in all cases, either sampling (≥4 lymph nodes removed) or level I-III dissection..

### Radiotherapy

Radiotherapy was given according to cancer centre local guidelines at the time. All patients who had undergone breast conserving surgery had post-operative radiotherapy. This consisted of the following (i) 50 Gy in 25 fractions to the breast over 5 weeks. In addition, those aged 50 years or under received an electron boost of 16 Gy in 8 fractions (ii) Following mastectomy and if more than 3 cam residual tumour, vascular invasion, or T4 cancer on presentation, chest wall irradiation (45 Gy in 20 fractions over 4 weeks) was carried out

If lymph nodes were involved by metastatic disease (assessed by sampling, level I/II dissection), the remaining axillary and supraclavicular lymph nodes were irradiated (45 Gy in 20 fractions over 4 weeks). If clearance was performed (level III) and lymph nodes were involved only the supraclavicular area was irradiated (45 Gy in 20 fractions over 4 weeks).

### Anti-hormonal

All patients with ER +ve and/or PR +ve breast cancers received tamoxifen 20 mg daily for 5 years.

### Quality of life

Before randomisation, and three weeks after completion of docetaxel, patients completed the Functional Assessment of Cancer Therapy-Breast (FACT-B) questionnaire (version 3) and the Rotterdam Symptom Checklist (RSCL) [20,21]. FACT-B assesses physical, social, emotional, functional wellbeing, and additional concerns specific to women with breast cancer. Patients indicated how true a statement had been for them over the past 7 days using a 5-point scale. High scores equate with a good QoL and low scores with a poor QoL. The RSCL yields separate measures for psychological and physical symptoms.

During docetaxel treatment (every 3 weeks), patients completed the Hospital Anxiety and Depression Scale (HADS), the Mood Rating Scale (MRS), the Global Distress Scale (GDS), and the Treatment Side-Effects Questionnaire (TSEQ) which measures the presence and severity of possible side-effects on a 5-point scale (not present; present but not distressing; slightly distressing, very distressing, and extremely distressing)[12,22-24].

### Clinical response

Tumour response was assessed during each cycle and on completion of chemotherapy by calliper and ultrasound measurements. Responses were graded according to the International Union Against Cancer criteria [25]; an absence of clinical evidence of tumour was classified as a complete clinical response (cCR); >50% reduction in the product of the two maximum perpendicular diameters of the tumour was classified as a partial clinical response (cPR); ≥25% increasing in size was classified as clinically progressive disease (cPD); clinical response that does not meet the definition of cCR, cPR, or cPD was classified as stable disease (cSD). Assessment of response after completion of NAC was made with reference to the size of tumour recorded prior to commencement of chemotherapy.

### Pathological response

Breast and axillary specimens were received fresh by the pathology department, thinly sliced and immersed in 10% neutral-buffer formalin for optimal fixation. All specimens were examined histologically using haematoxylin and eosin staining by a breast pathologist. Tumour response was evaluated using the Miller and Payne grading system [26]; grade 1, some alteration to individual malignant cells but no reduction in overall numbers as compared with the pre-treatment core biopsy; grade 2, a mild loss of invasive tumour cells but overall cellularity still high; grade 3, a considerable reduction in tumour cells up to an estimated 90% loss; grade 4, a marked disappearance of invasive tumour cells such that only small clusters of widely dispersed cells could be detected; grade 5, no invasive tumour cells identifiable in the sections from the site of the previous tumour (e.g., only in-situ disease or fibrosis remained). Metastatic nodes were assessed for pathological response (grade 1-5). Grade 5 response of the primary tumour represented a pCR.

### Outcome measures

The primary outcome was the Trial Outcome Index (TOI) of the FACT-B questionnaire; TOI is the sum of the scores from the physical, functional wellbeing, and breast cancer subscales (23 items in total) [27,28]. Higher TOI scores are associated with better QoL [29]. The secondary QoL outcomes were RSCL, HADS, MRS, GDS, and TSEQ.

Other outcomes were the clinical and pathological responses, incidence of BCS, DFS, and OS. DFS is the time from randomisation to local, regional, or distant treatment failure; occurrence of contralateral breast cancer; or death. OS is the time from randomisation to death from any cause.

### Statistical methods

Means and standard deviations of the TOI of FACT-B, obtained in our previous NAC study, suggested that a sample size of 40 in each group would have 80% power to detect a 6% difference in means using a two sample t-test with alpha at *p *= 0.05 (two-tailed) [12]. Allowance for the subject drop out was arbitrarily set at 15-20%. Therefore, it was necessary to recruit 95-100 patients into the trial.

Between-treatment differences on quality of life scales (FACT-B, RSCL, HADS, and MRS) at the primary endpoint (3 weeks after completion of chemotherapy) were analysed using Analysis of Covariance (ANCOVA) (with pre-randomisation values as covariate).

Because of non-normality of the distributions, between-treatment differences in GDS and each item of TSEQ were analysed for the primary endpoint (3 weeks after completion of chemotherapy) and the secondary endpoints (at 3, 6, and 9 weeks during docetaxel administration) using the Exact Probability-linear by linear test [30].

Comparisons of response outcomes between the treatment groups were analysed using the Chi-square test. Logistic regression analysis was performed to evaluate predictors of a pCR. Survival curves were estimated using the Kaplan-Meier method, and treatment comparisons were carried out using the log-rank test [31]. The Cox regression model was used to compute hazard ratios (HRs) and 95% confidence intervals (CIs), to examine the effect of prognostic variables, and to test for interactions between treatment and covariates. *P *values < 0.05 were considered statistically significant. Data were analysed using SPSS for Windows version 15.0. All those completing treatment were included in the analysis.

## Results

### Randomisation and enrollment

From July 2000 to November 2002, 96 patients were enrolled (Figure 1). One withdrew prior to commencing treatment; three withdrew before completion of AC (metastatic or progressive disease). Ninety-two patients completed four initial cycles of AC; three declined further chemotherapy, due to toxicity. Eighty-nine patients underwent treatment stratification, 45 were randomised to receive twelve further cycles of weekly docetaxel and 44 to receive four further cycles of 3-weekly docetaxel. Three patients declined further chemotherapy due to toxicity and two withdrew because of progressive disease. Two patients had metastatic disease after the third and fifth cycles of weekly docetaxel. Eighty-two patients (41 in each group) completed all cycles of chemotherapy and underwent surgery.

### Patient features and tumour characteristics

The groups were evenly matched for age, menopausal status, tumour size, tumour grade, clinical staging, and hormonal status (Table 1). The mean age was 49 years (range 27-70). Mean tumour calliper measurement was 4.2 cm (range 2.0-11.4). Over 80% of the cancers were T2 tumours. More than half were grade 3 and more than two-thirds expressed ER (>10% of cells).

**Table 1:** Patient features and tumour characteristics

Characteristic	Weekly docetaxel (n = 41)		3-weekly docetaxel (n = 41)		*P*-value
		
	**No**.	(%)	**No**.	(%)	
Age (years)					0.38
Mean	50.1		48.3		
Range	27-68		32-70		
Age at entry (years)					0.08
≤50	17	42	25	61	
>50	24	58	16	39	
Menopausal status					0.66
Premenopausal	22	54	24	58	
Postmenopausal	19	46	17	42	
Tumour size (cm) by caliper					0.41
Mean	4.1		4.3		
Range	2.0-6.2		2.5-11.4		
Tumour size (cm) by ultrasound					0.40
Mean	2.3		2.4		
Range	0.9-4.6		1.0-4.7		
Tumour stage					0.77
T1	1	2	0	0	
T2	32	79	35	86	
T3	7	17	5	12	
T4	1	2	1	2	
Tumour type					0.48
Invasive ductal	35	85	38	93	
Invasive lobular	6	15	3	7	
Tumour grade					0.21
1	5	12	1	2	
2	16	39	15	37	
3	20	49	25	61	
Clinical staging					0.39
IIA	22	53	15	37	
IIB	15	37	18	44	
IIIA	2	5	3	7	
IIIB	2	5	5	12	
Oestrogen receptor status					0.24
Positive	31	76	25	61	
Negative	10	24	15	37	
Unknown	0	0	1	2	
Progesterone receptor status					0.09
Positive	21	51	13	32	
Negative	13	32	23	56	
Unknown	7	17	5	12	

### QoL

#### Primary outcome

Adjusted mean scores for TOI and other secondary QoL outcomes are shown in Table 2. There were no significant differences in TOI scores between the weekly and 3-weekly groups at the primary endpoint (3 weeks after completion of chemotherapy) (*p *= 0.86). No differences in each subscale of FACT-B between groups were seen.

**Table 2:** Primary and secondary outcomes at three weeks after completion of chemotherapy

QoL scales	Weekly docetaxel	3-weekly docetaxel	F	*P*-value
			
	Adjusted mean (SE)	Adjusted mean (SE)		
FACT				
TOI	63.78 (1.66)	64.20 (1.68)	0.030	0.86
Physical	21.20 (0.73)	21.63 (0.74)	0.172	0.68
Social	22.79 (0.62)	23.23 (0.64)	0.241	0.63
Emotional	19.21 (0.51)	19.32 (0.51)	0.024	0.88
Functional	19.80 (0.71)	18.48 (0.72)	1.691	0.20
Additional	22.85 (0.59)	24.02 (0.60)	1.921	0.17
RSCL				
Physical	38.40 (1.14)	37.47 (1.14)	0.335	0.57
Psychological	12.79 (0.50)	11.76 (0.50)	2.057	0.16
HADS				
Anxiety	4.53 (0.44)	4.26 (0.44)	0.196	0.66
Depression	4.12 (0.39)	3.14 (0.39)	3.237	0.08
MRS				
Relaxation	88.59 (6.64)	104.49 (6.64)	2.708	0.10
Happiness	93.67 (6.29)	106.72 (6.29)	2.045	0.16
Energy	40.65 (6.12)	59.01 (6.12)	4.443	0.04
Clear-headedness	103.41 (5.90)	106.51 (5.90)	0.138	0.71
Easygoingness	94.63 (6.17)	94.37 (6.17)	0.001	0.98
Confidence	98.25 (6.07)	105.91 (6.07)	0.744	0.39
Total	548.27 (22.68)	637.64 (23.28)	7.101	0.01

Abbreviations: QoL, quality of life; SE, standard error; FACT, functional assessment of cancer therapy; TOI, trial outcome index; RSCL, Rotterdam symptom checklist; HADS, hospital anxiety and depression scale; MRS, mood rating scale.

#### Secondary outcomes

At the primary endpoint, there were no significant differences in RSCL, physical and psychological scores between groups. There were also no significant differences between groups in HADS anxiety and depression scores. Weekly group had significantly lower MRS total scores, compared with the 3-weekly group (*p *= 0.01); only MRS-energy subscale showed a significant difference (*p *= 0.04).

Of the 19 side-effects, only one was significant: neuropathy was present in 38.2% and rated significantly more distressing in the 3-weekly group (χ^2 ^= 7.205, *p *= 0.008). Overall, 39.5% rated their last 3 weeks treatment as 'not distressing'. However, distress (GDS) was significantly greater in the 3-weekly group (χ^2 ^= 4.99, *p *= 0.034).

Weekly docetaxel had significantly lower GDS scores at 3 weeks (χ^2 ^= 17.42.

*p *< 0.001), 6 weeks (χ^2 ^= 10.063, *p *= 0.001), and 9 weeks (χ^2 ^= 7.910, *p *= 0.005) during docetaxel administration. At least at one of these time points, patients in the weekly group had significantly less diarrhoea, constipation, nail problems, neuropathy, tiredness, pain, distress, depressed mood, and unhappiness.

### Clinical responses

The overall response rate (ORR: cCR + cPR) in 92 patients after four initial cycles of AC was 63% (Table 3). For the 82 patients who completed all cycles of docetaxel, ORRs were 93% in the weekly and 90% in the 3-weekly groups; 63% of patients in the weekly and 54% in the 3-weekly groups had achieved a cCR (*p *= 0.37). Sixty-five evaluable patients who did not achieve a cCR after AC, 58 (89%) were characterised as clinical responders after treatment with docetaxel.

**Table 3:** Clinical and ultrasound responses after completion of chemotherapy

Response	4AC (n = 92)		Weekly docetaxel (n = 41)		3-weekly docetaxel (n = 41)		*P*-value
		
	**No**.	%	**No**.	%	**No**.	%	
Clinical response							
cCR	17	18	26	63	22	54	0.70
cPR	41	45	12	29	15	37	
cSD	31	34	1	3	3	7	
cPD	1	1	2	5	1	2	
NA	2	2	0	0	0	0.0	
ORR	58	63	38	93	37	90	
US response							
cCR	16	19	12	29	12	29	0.71
cPR	37	40	21	51	17	41	
cSD	27	29	7	17	8	20	
cPD	2	2	0	0.0	0	0	
NA	9	10	1	3	4	10	
ORR	53	59	33	81	29	71	

Abbreviations: AC, doxorubicin+cyclophosphamide; cCR, clinical complete response; cPR, clinical partial response; cSD, clinical stable disease; cPD, clinical progressive disease; NA, not assessable; ORR, overall response rate (cCR+cPR); US, ultrasound

Ultrasonographic ORR of the tumour, in 92 patients after completion of AC, was 59%. For 82 patients randomised to weekly and 3-weekly docetaxel, ORRs were 81% and 71%, respectively (*p *= 0.30). The ultrasonographic cCR was equal (29%) in both groups.

### Pathological responses

In 82 patients, there was no significant difference in the pCR rate between groups (20% in the weekly and 27% in the 3-weekly groups, *p *= 0.43) (Table 4).

**Table 4:** Pathological responses and nodal status after completion of chemotherapy

Pathological response	Grade	Weekly docetaxel (n = 41)		3-weekly docetaxel (n = 41)		*P*-value
			
		**No**.	%	**No**.	%	
Primary tumour	1	10	24	7	17	0.90
	2	5	12	4	10	
	3	11	27	11	27	
	4	7	17	8	19	
	5	8	20	11	27	
Lymph node						
Negative		25	61	28	68	0.86
Positive	1	8	19	8	20	
	2	2	5	0	0	
	3	2	5	2	5	
	4	0	0	0	0	
	5	2	5	2	5	
	NA	2	5	1	2	

Abbreviation: NA, not assessable

Approximately two-thirds of patients in both groups had no evidence of nodal involvement. Of 29 patients having involved nodes, 16 (55%) had no evidence of nodal response following chemotherapy (grade 1); only 4 (14%) had complete response with scarring and fibrosis.

Using multivariate logistic regression analysis, none of the variables (age, clinical nodal status, and clinical tumour size) were statistically significant predictors of a pCR.

### Operative procedure

Almost half the patients (49% in the weekly and 42% in the 3-weekly groups) underwent BCS. Eight (20%) in the weekly and 5 (12%) in the 3-weekly groups required further surgery to achieve satisfactory clearance (*p *= 0.36). In patients with a cCR, 69% had BCS, compared with 27% without a cCR (*p *< 0.001). Thirty-four (83%) patients in the weekly and 39 (95%) in the 3-weekly groups underwent axillary sampling (*p *= 0.16). The rest of the patients, in both treatment groups, underwent axillary clearance.

### Compliance and toxicity

Six patients withdrew due to chemotherapy-related toxicity (three had AC only); mean number of chemotherapy cycles received by these patients was four.

There was no significant difference in the total intended drug dose of AC and docetaxel between groups. Five (6%) patients required a dose reduction of 25% or greater (AC, docetaxel); four patients required G-CSF support.

Of the 82 patients, only 5 (6%) experienced grade 3-4 neutropenia; 4 (5%) had febrile neutropenia only during AC administration. A higher percentage of patients in the 3-weekly groups experienced asthenia, neuropathy, peripheral oedema, and nail problems; the reverse was true for epiphora (Table 5). No death-related toxicity occurred during treatment.

**Table 5:** Treatment-related toxicity

Toxicity	Weekly docetaxel (n = 41)		3-weekly docetaxel (n = 41)		*P*-value
		
	**No**.	%	**No**.	%	
Nausea	3	7	5	12	0.71
Vomiting	3	7	2	5	1.00
Stomatitis	3	7	4	10	1.00
Asthenia	4	10	7	17	0.33
Neuropathy	0	0	16	38.2	0.008
Peripheral oedema	5	12	12	29	0.57
Epiphora (tearing)	13	32	8	20	0.21
Nail problems	9	22	15	37	0.15

### Survival

**Figure 2:**
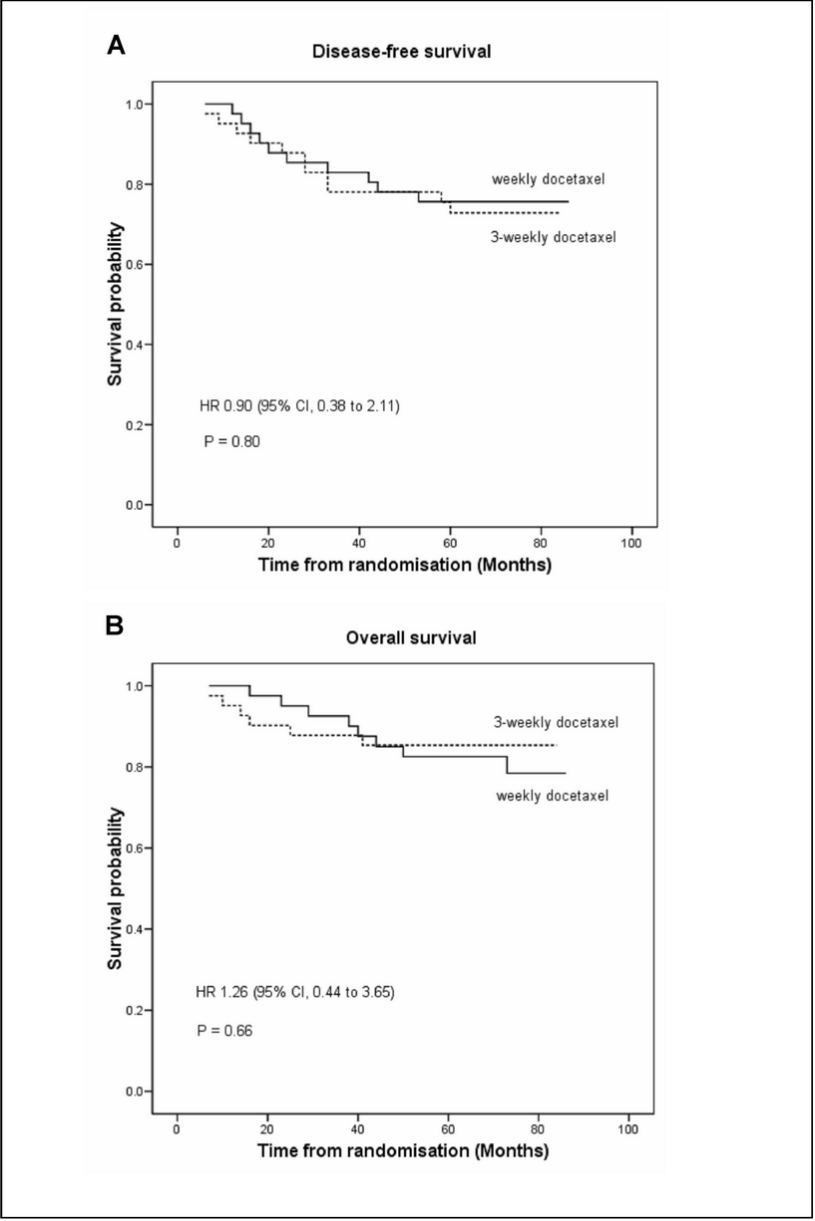
**Kaplan-Meier survival curves of (A) disease-free survival and (B) overall survival between treatment groups**. HR, hazard ratio; CI, confidence interval; (--), weekly docetaxel; (---), 3-weekly docetaxel

The median time on study with follow-up was 71.5 months. Of 82 patients, 21 (26%) experienced recurrences, of which 14 died (Table 6). The 5-year DFS were 76% in the weekly and 73% in the 3-weekly groups (*p *= 0.8). No difference in OS was seen between groups (81% in the weekly and 85% in the 3-weekly groups, *p *= 0.66) (Figure 2).

**Table 6:** Recurrences and death

Overall	Weekly docetaxel (n = 41)		3-weekly docetaxel (n = 41)		*P*-value
		
	**No**.	%	**No**.	%	
Recurrence	10	24	11	27	0.80
Locoregional	4	10	3	7	1.00
Contralateral	0	0	2	5	0.49
Distant	10	24	8	20	0.59
Death	8	20	6	15	0.49

**Figure 3:**
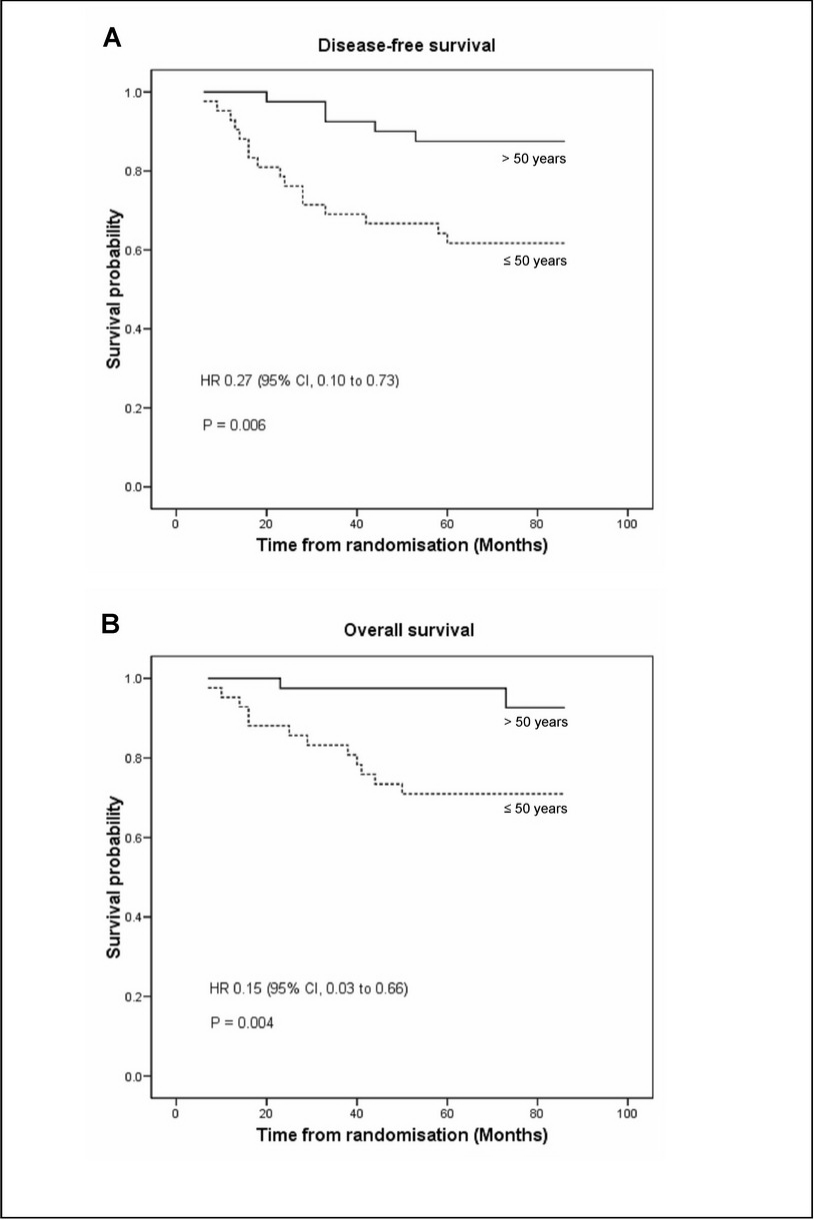
**Kaplan-Meier survival curves of (A) disease-free survival and (B) overall survival between age groups at study entry**. HR, hazard ratio; CI, confidence interval; (--), women >50 years; (---), women ≤50 years

Age at entry was a highly significant predictor of survival. Women >50 years had significantly better DFS (88% vs. 62%, *p *= 0.006) and OS (95% vs. 71%, *p *= 0.004), compared with those who were ≤ 50 years (Figure 3). These remained significant differences when adjusted for clinical tumour size, clinical and pathological nodal status, and tumour grade. The pCR and pathological nodal status were not predictors for survival in this study.

## Discussion

There has been an increasing interest in the use of NAC to downstage breast cancer and possibly increasing long-term survival. New drug combinations (antracyclines and taxanes) have been shown to be effective. Studies have suggested that the sequential use of docetaxel following AC is optimal. To improve efficacy and reduce morbidity, weekly taxanes have been used in patients with both advanced cancer and in the adjuvant setting. . The weekly regimen has demonstrated better tolerability, whilst maintaining efficacy comparable with the 3-weekly regimen[32,33].

To the best of our knowledge, this is the first report on QoL, psychosocial scores, and, concurrently, clinical and pathological responses of weekly versus 3-weekly docetaxel following pre-treatment with AC in the neoadjuvant setting. Three-weekly docetaxel is the standard regimen used in many centres treating patients with metastatic breast cancer. A weekly docetaxel regimen has not been shown to be superior, in a similar sized study to ours, in patients with metastatic breast cancer [34]. In a recently published radnomised controlled trial in women with metastic breast cancer, a weekly regimen (n = 77) compared with a 3 weekly regimen (n = 79) did not demonstrate a superior therapeutic outcome or quality of life [34]. However, a large meta-analysis combining 5 studies, comparing weekly versus 3 weekly regimens of paclitaxel in advanced breast cancer has clearly demonstrated better overall survival in the weekly regimen group (1471 patients, fixed effect model pooled HR 0.78, 95%CI 0.67-0.89 p = 0.001), as well as sigfnificantly reduced toxicity [35].

Our findings indicated that docetaxel, given weekly or 3-weekly, demonstrated similar generic measures of QoL (FACT-TOI, FACT-B, RSCL, HADS). The weekly group had lower distressing scores and reported less diarrhoea, constipation, nail problems, neuropathy, pain, tiredness, distress, depressed mood, and unhappiness. However, not all women preferred the weekly regimen, as it often interfered with a busy life schedule which probably explains the significantly lower energy and total MRS scores. Moreover, the weekly regimen requires more resources, thus, this need to be taken into consideration when planning the treatment regimens.

Our study has confirmed the results from other phase III studies of sequential docetaxel following anthracycline-based regimens. Two studies have evaluated weekly docetaxel only as NAC in stage II and III breast cancer [17,36]. In 56 patients, the ORR was 68%; 9 patients (16%) achieved a pCR. Another study randomised 913 patients with operable breast cancer to four cycles of preoperative doxorubicin combined with docetaxel every 2 weeks or four cycles of AC followed by four cycles of docetaxel every 3 weeks [11]. The sequential regimen had a higher ORR (85% vs. 75%; *p *< 0.001) and pCR rate (22% vs. 11%; *p *< 0.001). The weekly group in our study has shown an ORR of 93% and pCR rate of 20%, comparable with these results.

The pathological response within involved nodes was similar between the treatment groups. Only 14% of metastatic nodes showed a pCR. This result suggests that NAC is more effective in killing the primary tumour than the metastatic cells in the axillary nodes. Our study also documented a similar incidence of BCS in both groups.

The total intended drug dose and regimen duration in both groups were comparable. The weekly regimen was not associated with grade 3-4 neutropenia. All patients had a WHO performance status of 0, which may have contributed to the lower incidence of toxicity. A higher incidence of epiphora, as documented in our study, has been reported previously [37-39].

The Aberdeen study has shown that the addition of docetaxel to anthracycline-based NAC significantly increased the pCR rate and a 3-year DFS and OS [40]. However, the NSABP B-27 did not show a significant difference despite doubling the pCR rate [6]. In our study, the overall 5-year DFS and OS, irrespective of treatment schedule, were 74% and 83%, and the pCR rate was 23%. These results confirm the benefit of docetaxel with anthracycline-based regimens in NAC.

Several studies have shown that the pCR and pathological nodal status are predictors of long-term survival. Our study, however, did not confirm this, possibly due to the small cohort of patients. In this study, age at entry was a significant independent predictor for survival.

## Conclusions

Our small study confirms the benefits of using sequential weekly docetaxel in NAC for women with large or LABCs. Although the QoL was not significantly different, the lower distressing side-effect and the favorable toxicity profile may indicate the use of the weekly regimen for certain groups of patients, particularly the older patients or those with poor performance status. These beneficial effects require an increased number of clinic visits and more staff-time, but are not at the expense of clinical and pathological responses, BCS and survival outcomes.

## Competing interests

The authors declare that they have no competing interests.

## Authors' contributions

JME, MA, WV, MBW, ME, GC, JB, SS, JW, JAJ, DV, DC, MK, GAT, KB were involved with recruitment of patients, data acquisition and preparation of the manuscript. LGW and OE were involved with the design of the study, data interpretation, statistics, critical review of the manuscript and overall supervision of the work. All the authors read and approved the final manuscript.

## Pre-publication history

The pre-publication history for this paper can be accessed here:

http://www.biomedcentral.com/1471-2407/11/179/prepub
